# Right atrial rupture caused by primary cardiac angiosarcoma: A case report

**DOI:** 10.1097/MD.0000000000048459

**Published:** 2026-04-17

**Authors:** Haoxian Lai, Liya Wei

**Affiliations:** a Guangdong Medical University, Zhanjiang, China; b Department of Ultrasound lmaging, Peking University Shenzhen Hospital, Shenzhen, China.

**Keywords:** case report, color Doppler, primary cardiac angiosarcoma

## Abstract

**Rationale:**

Primary cardiac angiosarcoma is a rare malignant neoplasm of the heart. Clinical manifestations typically occur at an advanced stage, rendering early diagnosis challenging. Herein, we present the case of a young female patient with cardiac angiosarcoma complicated by atrial rupture.

**Patient concerns:**

The patient was a young woman who was admitted to the hospital after a sudden episode of syncope with loss of consciousness. Transthoracic echocardiography revealed a solid mass located in the anterolateral wall of the right atrium, accompanied by a large amount of pericardial effusion. Computed tomography and magnetic resonance imaging findings were suggestive of cardiac angiosarcoma. Consequently, the patient underwent resection of the malignant cardiac tumor and repair of cardiac rupture. Histopathological examination demonstrated tumor cells with abundant eosinophilic cytoplasm. Immunohistochemical analysis showed that the tumor cells were positive for CD31 and ERG.

**Diagnoses:**

Based on the clinical presentation, imaging findings, and immunophenotypic evidence, the patient was diagnosed with primary cardiac angiosarcoma.

**Interventions:**

The patient received regular postoperative chemotherapy.

**Outcomes:**

After regular follow-up for 8 months, the patient was transferred to continue chemotherapy and died due to multiple metastatic tumors.

**Lessons:**

Primary cardiac angiosarcoma is a rare malignant cardiac tumor. Because clinical manifestations usually occur at an advanced stage, early diagnosis remains challenging. The comprehensive use of multiple imaging modalities is beneficial for accurate lesion identification, tumor staging, and thorough disease evaluation.

## 1. Introduction

Primary cardiac angiosarcoma (PCAS) is a malignant tumor arising from the endothelial cells of cardiac blood vessels. Malignant tumors constitute approximately 20% of primary cardiac tumors, with angiosarcoma being the most common pathological subtype, accounting for approximately 28.6% of these cases.^[1]^ Because the clinical manifestations of cardiac angiosarcoma are often nonspecific, diagnosis typically occurs at an advanced stage, resulting in a poor prognosis. The average age at diagnosis is approximately 46 years, with no significant difference in incidence between males and females. The median survival time is approximately 25 months. For patients without metastasis, complete surgical resection remains the preferred treatment to achieve optimal survival outcomes.^[2]^

## 2. Case presentation

The patient was a female who presented with sudden syncope, loss of consciousness, and shortness of breath. After receiving external chest compressions, she was transferred to the emergency department of our hospital. On arrival, she was found to have cold and clammy skin and was immediately moved to the emergency resuscitation room for further management. Initial assessment revealed a heart rate of 135 bpm, unmeasurable blood pressure, impaired consciousness, cyanotic lips, generalized cold and clammy skin, and diminished heart sounds. Fluid resuscitation, epinephrine, and dopamine were administered to restore hemodynamic stability. Bedside echocardiography revealed a moderate to large pericardial effusion. Emergency pericardiocentesis with catheter placement was performed, draining approximately 180 mL of hemorrhagic pericardial effusion, and a drainage tube was left in situ. Following the procedure, the patient’s heart rate decreased to 70 to 80 bpm, and her blood pressure increased to 112/79 mm Hg. She was subsequently admitted to the intensive care unit for further management. Continuous subxiphoid pericardial drainage yielded dark red hemorrhagic fluid. Cytopathological examination of the pericardial effusion revealed no definitive malignant cells.

Echocardiography demonstrated a large anechoic area within the pericardial cavity causing right heart diastolic collapse. The heart appeared to swing within the effusion, and the inferior vena cava showed impaired venous return. A moderately echogenic, irregularly shaped mass with well-defined margins and heterogenous internal echoes was visualized in the anterolateral wall of the right atrium. The mass measured approximately 54 mm × 44 mm, with an indistinct interface between the mass and the pericardium. Color Doppler flow imaging showed no definite blood flow signal within the lesion. The inferior vena cava was dilated (approximately 23 mm in diameter) with a respiratory collapse rate of <50%. Conclusion: A solid mass was detected in the anterolateral wall of the right atrium, with indeterminate characteristics. Further evaluation was recommended. The inferior vena cava was dilated, while left ventricular systolic function remained normal.

**Figure 1.**
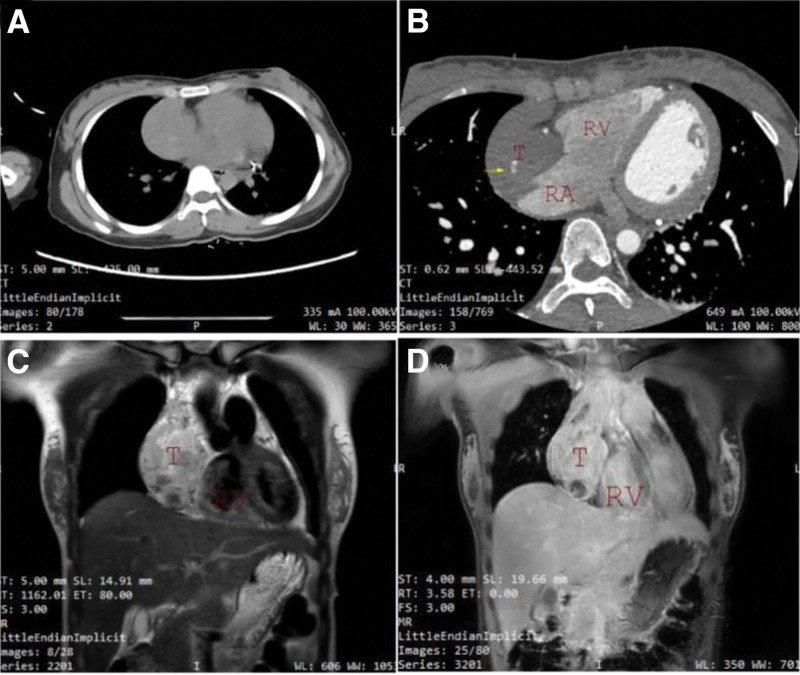
(A) Non-contrast CT (computed tomography) shows a large, slightly hyperdense, poorly homogeneous mass in the right atrium. (B) Contrast-enhanced CT reveals minimal contrast uptake (yellow arrow) with limited enhancement. (C) Non-contrast MRI demonstrates the mass broadly attached to the right atrial wall, showing a heterogeneous, slightly hyperintense signal on T2WI. (D) Postcontrast MRI shows marked heterogeneous enhancement with progressive contrast uptake and small patchy nonenhancing low-signal areas. CT = computed tomography, MRI = magnetic resonance imaging, RA = right atrium, RV = right ventricle, T = tumor.

Non-contrast chest CT combined with chest aortic computed tomography angiography (CTA) demonstrated a large, lobulated mass-like lesion of slightly increased attenuation located on the right side of the heart. The lesion showed heterogeneous density with a mean attenuation of approximately 50 HU on non-contrast CT, measuring about 51 mm × 59 mm × 60 mm. The margin between the lesion and the right atrium was indistinct. On contrast-enhanced imaging, a small amount of contrast agent was observed to enter the lesion. Conclusion: A large lobulated mass-like lesion of slightly increased attenuation in the right pericardial region with minimal contrast enhancement, suggestive of tumor rupture with associated hemorrhage (Fig. 1A, B).

Cardiac magnetic resonance imaging (MRI) revealed an irregular mass in the anterolateral wall of the right atrium, broadly attached to the atrial wall. The right atrial wall appeared undulated, and the superior vena cava was compressed. The mass measured approximately 6.5 cm × 5.6 cm × 12.4 cm. Contrast-enhanced imaging showed marked heterogeneous enhancement with progressive contrast uptake, and small patchy nonenhancing low-signal areas were observed within the lesion. Conclusion: A malignant mesenchymal tumor was suspected, with angiosarcoma considered the most likely diagnosis (Fig. 1C, D) . Subsequently, the patient underwent resection of the malignant cardiac tumor, cardiac rupture repair, and closure of a patent foramen ovale at our hospital.

Intraoperatively, fibrin deposits were observed on the cardiac surface, and a mass was identified in the right atrium. The lesion measured approximately 6 cm × 5 cm. The upper margin was near the junction of the superior vena cava and the right atrium, the lower margin was approximately 2 cm from the opening of the inferior vena cava, the posterior margin was approximately 1.5 cm from the interatrial groove, and the anterior margin encased the right coronary artery. No evident invasion of the right ventricular wall was noted. The surface of the mass was cauliflower-like in appearance, and a spontaneous rupture measuring approximately 0.5 cm in diameter was present at its center. These findings strongly indicated a malignant cardiac tumor. The thickest portion of the lesion measured approximately 2 cm, with thrombus formation observed internally. Intraoperative frozen section analysis could not exclude the possibility of a right atrial sarcoma.

### 2.1. Postoperative pathological report

#### 2.1.1. Gross findings

The specimen labeled “right atrial mass” consisted of multiple fragments of gray-red tissue, with a total size of approximately 6 cm × 5 cm × 3 cm and an ill-defined structure. The surgical margins could not be evaluated. Part of the specimen was covered by a suspected capsule measuring approximately 7 cm × 4 cm.

#### 2.1.2. Microscopic findings

**Figure 2.**
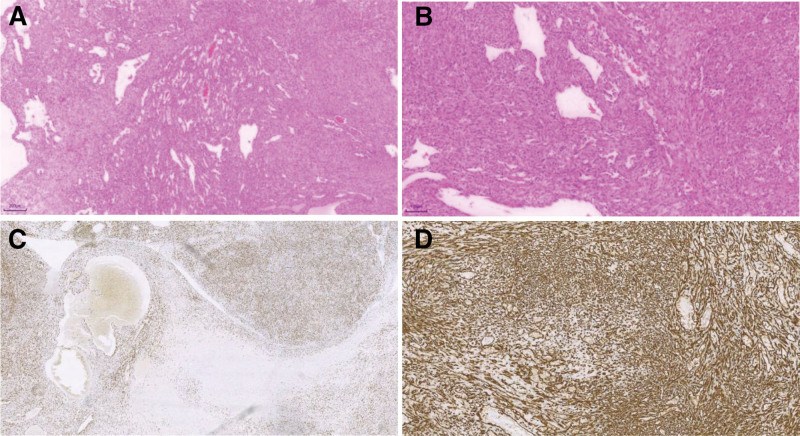
(A, B) Tumor tissue composed of numerous slit-like or dilated vascular channels, occasionally containing intravascular tumor thrombi. Perivascular spindle-shaped tumor cells proliferate in streaming or interlacing patterns, with extravasated erythrocytes observed between the cells. The tumor cells have abundant pale eosinophilic cytoplasm and round to oval nuclei with fine chromatin, and mitotic figures are readily visible (hematoxylin and eosin staining: A, ×5; B, ×10). (C, D) Immunohistochemistry photomicrographs of CD31 (C), ERG (D) stain (C, ×20; D, ×48). CD31 = cluster of differentiation 31, ERG = E‑twenty-six‑related gene.

The tumor was composed of numerous slit-like or dilated vascular channels, with occasional intravascular tumor thrombi. Proliferation of spindle-shaped tumor cells was observed around the vascular spaces, arranged in streaming or interlacing patterns, with extravasated red blood cells scattered among the tumor cells. The neoplastic cells exhibited abundant pale eosinophilic cytoplasm and round to oval nuclei with fine chromatin. Mitotic figures were readily observed. In certain areas, invasion into cardiac muscle fibers was evident (Fig. 2A, B).

#### 2.1.3. Pathological diagnosis

“Right atrial mass” – malignant spindle cell tumor consistent with angiosarcoma.

### 2.2. Immunohistochemical results of spindle tumor cells (with appropriate positive and negative controls)

CD31 (+), CD34 (+), ERG (+), SMA (−), Desmin (−, indicating invasion into cardiac muscle fibers), Ki-67 (hotspot area approximately 40%+), HHV-8 (−), INI-1 (+), CK (pan) (−; Fig. 2C, D).

### 2.3. Follow-up findings

Postoperative echocardiography showed the patient’s status post resection of a malignant cardiac tumor, cardiac rupture repair, and closure of a patent foramen ovale. No shunt was detected at the atrial level. Left ventricular systolic and diastolic functions were normal. Chest CT revealed no visible soft tissue mass in the region of the previously identified right atrial lesion. Genetic testing identified mutations in the DRR and ATM genes, and the patient was subsequently treated with niraparib.

After 1 month of treatment, follow-up echocardiography demonstrated a slightly hypoechoic mass measuring approximately 44 mm × 43 mm in the lateral wall of the right atrium. The mass appeared relatively regular in shape, with an indistinct boundary and heterogeneous internal echogenicity.

**Figure 3.**
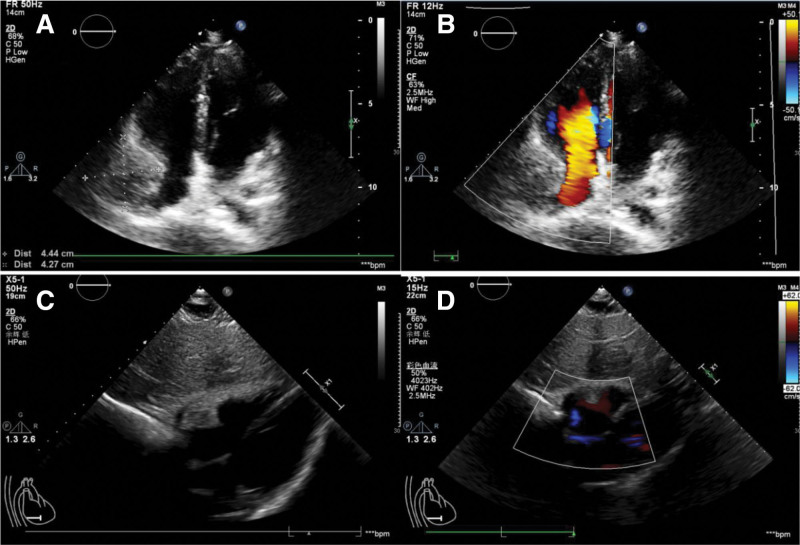
(A) One-month postoperative cardiac echocardiogram showing a slightly hypoechoic mass measuring approximately 44 × 43 mm on the lateral wall of the right atrium. The mass has a relatively regular shape, ill-defined borders, and heterogeneous internal echogenicity. (B) Color Doppler imaging reveals no significant blood flow signal within the mass. (C, D) A slightly hypoechoic mass is observed at the anterolateral aspect of the pericardium on the subxiphoid view, with compression of the superior vena cava and the right atrium. The margins of the lesion are ill-defined.

A solid mass was detected in the lateral wall of the right atrium, warranting further evaluation. No residual shunt was detected (Fig. 3).

Follow-up non-contrast cardiac CT revealed a mildly hyperdense, mass-like shadow in the anterolateral pericardial region. The lesion exhibited heterogeneous density and measured approximately 46 mm × 38 mm × 41 mm. The superior vena cava and right atrium were compressed, and the mass displayed an irregular shape with an indistinct boundary between it and the right atrium. Contrast-enhanced imaging revealed multiple tortuous small vascular structures within the lesion, showing heterogeneous enhancement.

Postsurgical changes of the heart were noted, with a mass in the anteroinferior pericardial region that had significantly increased in size compared with the previous examination. The indistinct boundary between the lesion and the right atrium suggested tumor recurrence.

PET–CT (positron emission tomography–computed tomography) showed postoperative changes in the right atrial region. An irregular mass was identified within and adjacent to the right atrial appendage, with a standardized uptake value of 8.7, consistent with recurrent tumor.

After 3 months of albumin-based chemotherapy, echocardiography showed a right atrial lateral solid position that smaller than in the previous examination. Chest CT indicated a soft tissue mass at the right margin of the right atrium, with a maximum size of approximately 37 mm × 19 mm and a CT attenuation of approximately 24HU, significantly smaller than at 2 months after surgery.

The patient continued to undergo regular follow-up for 8 months postoperatively, during which the tumor size remained largely unchanged. She was later transferred to Peking University Cancer Hospital and Peking Union Medical College Hospital for further treatment. Despite continued chemotherapy, the patient eventually died due to multiple metastases of the tumor (specific details unknown).

## 3. Discussion

Primary cardiac angiosarcoma is a rare malignant mesenchymal neoplasm and represents the most common histologic subtype of primary malignant cardiac tumors. However, its clinical manifestations are often nonspecific, making early diagnosis challenging and requiring a high index of clinical suspicion. Echocardiography is the preferred initial imaging examination for diagnosis, although confirmation ultimately depends on pathological examination. Early manifestations of PCAS are atypical, with pericardial effusion and right heart failure being the most common initial symptoms.^[2,3]^ In this case, the patient’s first manifestation was cardiac rupture and pericardial tamponade caused by tumor invasion.

Primary cardiac angiosarcoma most frequently arises in the right heart, particularly in the lateral wall of the right atrium, followed by the right atrioventricular groove and the extra-pericardial region of the right atrium.^[4]^ Echocardiography plays a vital role in tumor detection. The typical echocardiographic appearance is an irregular, solid intracavitary mass with infiltrative growth into the myocardium, a broad-based attachment, and limited mobility. Two-dimensional echocardiography provides essential information on the lesion’s location, size, morphology, and mobility.^[5,6]^ Approximately 56% of patients with PCAS present with pericardial effusion at diagnosis, although most do not exhibit significant effusion.^[7]^ When pericardial effusion is present, ultrasound sensitivity for detecting extra-cavitary tumors decreases.^[8,9]^ In cases of large pericardial effusion, cardiac tamponade may occur, compressing the atrioventricular wall and obscuring tumor visualization. Therefore, patients presenting with isolated pericardial effusion should be closely monitored to rule out cardiac angiosarcoma. Even in the absence of malignant cells in the pericardial fluid, PCAS cannot be excluded, underscoring the diagnostic challenge.^[10,11]^ Determining whether a mass is pedunculated is crucial for differentiating benign tumors such as myxomas or papillary fibroelastomas. Doppler echocardiography can also assess hemodynamic alterations caused by tumor obstruction. Vascular sarcomas often arise in the right atrioventricular groove, predisposing the tricuspid valve to invasion and causing obstruction in the right ventricular outflow tract.^[11]^ Clinical symptoms such as dyspnea and chest tightness further support hemodynamic compromise.^[12]^

In clinical practice, echocardiography and CT are routinely used to detect intracardiac masses and assess preoperative anatomy.^[13,14]^ Cardiac MRI offers superior-tissue characterization, greater sensitivity for detecting intratumoral hemorrhage, and better delineation of tumor margins relative to adjacent myocardium.^[15]^ Therefore, multimodality imaging provides a comprehensive assessment of tumor morphology, extent, and surgical resect-ability.

Surgical excision remains the cornerstone of treatment for localized PCAS. Compared with monotherapy (surgery or chemotherapy alone), a multimodal approach combining neoadjuvant chemotherapy and surgical resection has been shown to improve prognosis.^[16]^ Reported median survival is approximately 14.1 months with single-modality therapy versus 36.5 months with combined treatment.^[17]^ Some case reports suggest that integrative approaches combining traditional Chinese and Western medicine may also provide partial clinical benefits, indicating potential value in improving patient outcomes.^[18]^

Primary cardiac angiosarcoma must be differentiated from various benign and malignant cardiac tumors, thrombi, metastatic lesions, and infective vegetations.^[19]^ Benign tumors such as cardiac myxomas are typically pedunculated, attached to the interatrial septum, and move between the atrium and ventricle during the cardiac cycle. Malignant tumors, in contrast, require more detailed diagnostic evaluation based on anatomic location, morphology, and clinical context. For example, primary cardiac lymphoma, which also commonly arises in the right atrium, typically appears as a lobulated or poorly defined mass with a broad-based attachment to the myocardium. However, unlike angiosarcoma, primary cardiac lymphoma rarely exhibits calcification or necrosis, and only small focal or linear blood flow signals are usually within the lesion.

In this case, the patient showed typical manifestations of primary cardiac angiosarcoma, including massive pericardial effusion and pericardial pressure. Subsequently, tumor invasion led to cardiac rupture – a rare and life-threatening complication of angiosarcoma. Throughout the diagnostic and treatment process, echocardiography, CT, MRI, and PET/CT were employed to assess the preoperative lesion and monitor postoperative recurrence. Because of the insidious nature of cardiac vascular sarcoma, early detection and diagnosis remain challenging. Therefore, the integrated use of multiple imaging modalities is essential for accurate lesion identification, staging, and comprehensive disease evaluation.

## Author contributions

**Data curation:** Haoxian Lai.

**Supervision:** Liya Wei.

**Writing – original draft:** Haoxian Lai.

**Writing – review & editing:** Liya Wei.
